# Current intraoperative storage and handling practices of autologous bypass conduit: A survey of the royal australasian college of surgeons

**DOI:** 10.3389/fsurg.2022.956177

**Published:** 2022-08-26

**Authors:** AB Haymet, N Pinto, S Peden, T Cohen, MP Vallely, D McGiffin, R Naidoo, J Jenkins, JY Suen, JF Fraser

**Affiliations:** ^1^ Department of Vascular Surgery, The Royal Brisbane and Women’s Hospital, Herston, QLD, Australia; ^2^ Critical Care Research Group, The Prince Charles Hospital, Chermside, QLD, Australia; ^3^ Faculty of Medicine, University of Queensland, St Lucia, QLD, Australia; ^4^ Herston Biofabrication Institute, Royal Brisbane and Women’s Hospital, Herston, QLD, Australia; ^5^ Department of Vascular Surgery, The Princess Alexandra Hospital, Woolloongabba, QLD, Australia; ^6^ Department of Cardiovascular Surgery, Mount Sinai Morningside/Icahn School of Medicine, New York, NY, United States; ^7^ Department of Cardiothoracic Surgery, The Alfred Hospital, Melbourne, VIC, Australia; ^8^ Faculty of Medicine, Monash University, Melbourne, Australia; ^9^ Department of Cardiothoracic Surgery, The Prince Charles Hospital, Chermside, QLD, Australia

**Keywords:** conduit, bypass, graft, intraoperative, RACS = royal australasian college of surgeons, early graft failure

## Abstract

During bypass surgery for peripheral arterial occlusive disease and ischaemic heart disease, autologous graft conduit including great saphenous veins and radial arteries are frequently stored in solution. Endothelial damage adversely affects the performance and patency of autologous bypass grafts, and intraoperative graft storage solutions have been shown to influence this process. The distribution of storage solutions currently used amongst Cardiothoracic and Vascular Surgeons from Australia and New Zealand is not well defined in the literature. The aim of this study was to determine current practices regarding autologous graft storage and handling amongst this cohort of surgeons, and discuss their potential relevance in the context of early graft failure. From this survey, the most frequently used storage solutions were heparinized saline for great saphenous veins, and pH-buffered solutions for radial arteries. Duration of storage was 30–45 min for almost half of respondents, although responses to this question were limited. Further research is required to investigate whether ischaemic endothelial injury generates a prothrombotic state, whether different storage media can alter this state, and whether this is directly associated with clinical outcomes of interest such as early graft failure.

## Introduction

During bypass surgery for peripheral arterial occlusive disease and ischaemic heart disease, autologous graft conduit including great saphenous veins (GSV) and radial arteries (RA) are frequently stored in solution prior to anastomosis. Early graft failure (EGF), defined as occlusion within 30 days (1), remains a serious postoperative surgical complication, with rates of 4.5% and 3% previously reported for infrainguinal and coronary bypass graft procedures, respectively (2, 3). Data reporting the most frequently used storage solutions, as well as intraoperative handling practices (i.e. use of “no touch” techniques” where the vein is harvested with a pedicle of surrounding tissue (4), and the extent to which the vein is mechanically distended) amongst Cardiothoracic and Vascular surgeons in Australia and New Zealand are lacking. Previous studies have suggested that storage media can influence conduit endothelial injury, and influence early graft patency (5). However, this is an area which is currently in need of further research (6). The aim of this study was to determine current practices regarding autologous graft storage and handling amongst surgeons in Australia and New Zealand, and discuss their potential relevance in the context of EGF.

## Methods

Institutional Review Board approval was obtained (ID 69579, The Prince Charles Hospital HREC (EC00168), 22 October 2020). An electronic survey was distributed online *via* the Cardiothoracic and Vascular divisions of the Royal Australasian College of Surgeons using SurveyMonkey (San Mateo, CA, United State of America). Responses were anonymous and not identifiable. The survey questions are available in the Appendix.

## Results

**Figure 1:**
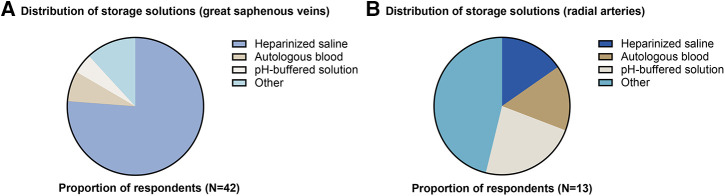
Distribution of storage solutions used for (**A**) great saphenous veins and (**B**) radial arteries.

43 responses were received from 395 members approached (response rate = 10.9%). Response rate for Cardiothoracic and Vascular divisions was 8.3% (12/145) and 12.4% (31/250), respectively. 93% of respondents (*n* = 40) were currently practicing. All respondents (100%) routinely harvested GSV, and 33.3% routinely harvested RA. For GSV, storage solutions used were heparinized saline (76.2%), “other” (11.9%), autologous blood (7.1%), or pH-buffered solution (4.8%) (Figure 1A). “Other” solutions included heparinized saline and papaverine (7.1%), autologous blood and verapamil (2.4%) or no storage at all (2.4%). For RA, storage solutions used were “other” (46.1%), pH-buffered solution (23.1%), autologous blood (15.4%) and heparinized saline (15.4%) (Figure 1B). For “other” solutions, these included heparinized blood and verapamil (15.4%), glyceryl trinitrate and verapamil (7.7%), papaverine solution to the extraluminal vessel only (7.7%), heparinized blood with papaverine (7.7%), or heparinized blood with diltiazem and glyceryl trinitrate (7.7%). Specialty-specific responses regarding storage solution, duration, “no-touch” harvesting and extent of conduit distension are shown in Table 1.

**Table 1:** Distribution of storage solutions, storage duration, “no-touch” harvesting and degree of conduit distension by surgical specialty^a^.

		Vascular Surgery	Cardiothoracic Surgery	Total
Storage solutions for harvested great saphenous veins	Heparinized saline	25	7	32
		17.99%	5.04%	
		78.13%	21.88%	
		83.33%	58.33%	
	Autologous blood	1	2	3
		0.72%	1.44%	
		33.33%	66.67%	
		3.33%	16.67%	
	pH- buffered solution	0	2	2
		0%	1.44%	
		0%	100%	
		0%	16.67%	
Storage solutions for harvested radial arteries	Heparinized saline	1	1	2
		0.72%	0.72%	
		50%	50%	
		3.33%	8.33%	
	Autologous blood	0	2	2
		0%	1.44%	
		0%	100%	
		0%	16.67%	
	pH- buffered solution	0	3	3
		0%	2.16%	
		0%	100%	
		0%	25%	
Average storage duration of harvested conduits	Less than 5 min	1	0	1
		0.72%	0%	
		100%	0%	
		3.33%	0%	
	5 to 15 min	0	2	2
		0%	1.44%	
		0%	100%	
		0%	16.67%	
	15 to 30 min	0	4	4
		0%	2.88%	
		0%	100%	
		0%	33.33%	
	30 to 45 min	0	6	6
		0%	4.32%	
		0%	100%	
		0%	50%	
“No-touch” technique used to harvest the vein or artery (i.e. the vein or artery is harvested with a pedicle of surrounding tissue)	Yes	9	8	17
		6.47%	5.76%	
		52.94%	47.06%	
		30%	66.67%	
	No	20	4	24
		14.39%	2.88%	
		83.33%	16.67%	
		66.67%	33.33%	
Extent of mechanical distension the conduit following harvest:	None	0	1	1
		0%	0.72%	
		0%	100%	
		0%	8.33%	
	Enough to fill the conduit but not expand it beyond its native (pre-dissection) diameter	12	8	20
		8.63%	5.76%	
		60%	40%	
		40%	66.67%	
	The conduit expands up to 1.5-2x its native (pre-dissection) diameter	16	3	19
		11.51%	2.16%	
		84.21%	15.79%	
		53.33%	25%	
	The conduit expands more than 2x its native (pre-dissection) diameter.	1	0	1
		0.72%	0%	
		100%	0%	
		3.33%	0%	

^a^ Data reported as Count, Percent of total, Row percentage, Column percentage*.*

**Figure 2:**
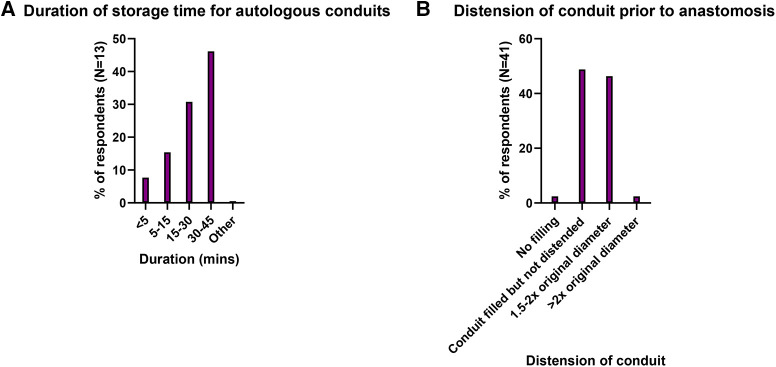
(**A**) Distribution of storage times for harvested autologous conduits and (**B**) Distension of conduit prior to anastomosis.

Storage duration for these conduits was 30–45 min in 46.2% (6/13) (Figure 2A). A “no-touch” technique was used in 41.5% (17/41). Mechanical distension of the conduit was beyond twice the native vessel diameter in 2.4%, 1.5–2 times native diameter in 46.3%, filled but not distended in 48.8%, and no distension in 2.4% (Figure 2B).

## Discussion

The present study aimed to identify current intraoperative conduit storage practices in Australia and New Zealand, and discuss their potential relevance with respect to EGF, which remains a substantial problem in cardiovascular surgery (2, 3). From this survey, for great saphenous veins, heparinized saline was clearly the predominant storage solution. For radial arteries, which are of primary relevance to cardiothoracic surgeons in Australia and New Zealand as a second arterial conduit following an internal thoracic arterial graft (7), storage solutions were more diversely spread across heparinized saline, autologous blood, buffer solutions and modified buffer solutions with vasodilators. This may potentially suggest a lack of consensus with respect to radial artery storage. Storage time was up to 45 min in almost half of respondents, although responses to this question were limited. It was also noted that the response rate to the survey was relatively low, which must be borne in mind when interpreting the findings.

The effect of intraoperative graft storage solutions on endothelial damage, potential upregulated thrombogenicity, and potential subsequent graft failure is worthy of discussion. The PREVENT IV trial identified that in 2,817 patients who underwent CABG, veins stored in saline or blood-based solutions demonstrated a higher rate of failure at one year compared to buffered saline (5). Previously suggested reasons for saline being harmful to endothelium include its lack of ionic balance, and its acidic pH (5.5) (8).

Thrombosis is the major cause of early graft failure (9). Whilst thrombogenicity in this setting is highly multifactorial, endothelial injury of the conduit arguably plays an important role, and intraoperative storage solutions influence this process (5, 8, 10). Structurally, the endothelial surface layer is defined as the endothelial cellular glycocalyx, which is a layer of glycans lining all human cells, and its associated plasma proteins (11–13). The glycocalyx is a matrix consisting of various proteoglycans, glycosaminoglycans (GAGs), and plasma proteins, and it provides endothelial cellular mechano-sensation and transduction (14). Its principal GAGs include heparan sulphate (HS) and hyaluronic acid (HA), and core proteins primarily include syndecans and glypicans (11). Damage to the endothelial cell glycocalyx appears to be the earliest detectable injury to the vascular wall during the development of atherosclerosis and is associated with increased vascular permeability and adhesiveness (15). Destruction of the endothelial glycocalyx, which ranges from 200 to 2000nm in thickness, decreases vascular barrier function and leads to protein extravasation and tissue oedema, loss of substrate supply to tissues, and an increase in platelet and leucocyte adhesion (16).

During surgery, early endothelial injury begins during conduit preparation, including the harvesting technique used (e.g. open versus endoscopic), the extent to which the graft is manipulated and distended (17, 18), and surgical technique used during anastomosis. This is reflected by the teaching of “no touch” or minimal graft handling techniques, minimizing the over-distension of bypass conduits, and meticulous attention to anastomoses (19). It is worth noting that in certain circumstances, such as in-situ infrainguinal bypass surgery, storage of a free graft will be obviated, and periodic flushing is often employed in this setting once a proximal anastomosis has been completed. Endothelial damage, such as mechanical de-endothelialization, is frequently observed in free saphenous vein grafts (20) and exposes the underlying extracellular matrix. This triggers local release of tissue factors with reduced bioavailability of prostacyclin and nitric oxide (NO), which culminates in enhanced platelet activation, fibrin deposition, and ultimately thrombosis (21). During conduit harvesting, the endothelium is also rendered ischaemic due to separation from the systemic circulation and disruption of vasa vasorum of the vessel wall. Ischaemia generates oxidative stress, which may activate a procoagulant state (22). Luminal expression of prothrombotic molecules, such as thromboxane A2 and plasminogen activator inhibitor-1 upregulates the interaction between an activated endothelial surface with platelets and leucocytes. This sets in motion an accelerating process of inflammation and thrombosis, and ultimately, graft thrombosis (23).

The endothelial expression of thromboprotective proteins, such as thrombomodulin, plays a vital role in early graft patency (9). Thrombomodulin is a surface glycoprotein which modulates the activity of thrombin from a procoagulant to an anticoagulant protease (24), and its expression is vital in graft thromboresistance. When bound to thrombomodulin on the endothelial surface, thrombin is unable to generate fibrin or activate platelets but instead becomes a potent activator of protein C. The activated form of protein C (APC) is an anticoagulant protease that selectively inactivates coagulation factors Va and VIIIa, providing an essential feedback mechanism to prevent excessive coagulation. Although activation of protein C *in vivo* is completely dependent on thrombomodulin, the efficiency of protein C activation is enhanced by another endothelial cofactor, the endothelial protein C receptor (EPCR) (25). Furthermore, ischaemic injury has been shown to downregulate thrombomodulin expression (26). Kim *et al* demonstrated, in a rodent model, that early loss of TM expression significantly impairs vein graft thromboresistance and results in enhanced local thrombin generation (9). Immunohistochemical staining of autologous rabbit vein graft sections revealed that the expression of TM, but not EPCR, was reduced significantly early after graft implantation. Western blot analysis revealed that TM expression was reduced by >95% during the first 2 weeks after implantation, with gradual but incomplete recovery by 42 days (9).

Despite the clinical burden of acute conduit occlusion, whilst some studies have previously investigated the influence of different storage solutions on endothelial integrity, few have investigated their effect on thrombogenesis (27). In order to mitigate endothelial shedding secondary to ischaemic injury, as well as the prothrombotic and proinflammatory state which accompanies it, a small number of novel treatment solutions have been studied *in vitro* and *in vivo.* Normal saline, whilst extensively used as a graft storage solution, has been shown to be damaging to autologous grafts, demonstrated both histologically as well as functionally, with impaired endothelial-dependent vasoreactivity (28, 29). Cardioplegia is used for myocardial protection during cardiac surgery. Generally, they may be classified as blood or crystalloid forms, such as St Thomas', del Nido, and Bretschneider solutions. Crystalloid cardioplegia was initially used to achieve myocardial protection until Buckberg introduced the concept of blood-based cardioplegia, which subsequently became increasingly popular (30). With respect to graft conduit storage, a recent prospective trial by Papakonstantinou *et al* reported that cardioplegia may better protect endothelial cells compared to heparin enriched solutions, however the association with clinical outcomes remains to be proven (31).

Furthermore, a new chloride-poor, iron-chelator-enhanced cardioplegic solution (Custodiol-N) has demonstrated improved liver, lung and heart preservation in different experimental studies (32–34). In a large animal study by Veres *et* al., this novel (Custodiol-N) conferred greater coronary endothelial protection compared to Custodiol after hypothermic cardiac arrest (35). TiProtec, a chloride-depleted, iron chelator-fortified modified HTK solution and Duragraft, an endothelial damage inhibitor, have shown promising results in preclinical studies involving both in murine aortic tissue and human saphenous veins. In a recent study in 2016, Veres *et al*. reported that in a murine model where aortic arches were harvested, stored in a novel TiProtec preservation solution, and grafted to the abdominal aorta, endothelial function was better preserved in the TiProtec group when compared with the saline and Custodiol groups (35). In a study of human saphenous vein segments and isolated pig mammary veins by Pachuk *et al*., normal saline caused damage to vascular endothelium, loss of graft cell viability, and mediated cell damage, whereas no evidence of damage or reactivity was observed in DuraGraft-exposed cells (29).

It is justifiable that the conduit endothelium should be protected as much as possible from ischaemic injury from the moment it is harvested. Intraoperative storage solutions may influence this pathophysiological process. Further research is required, however, regarding the effect of intraoperative storage media on expression of thromboprotective proteins, such as thrombomodulin (27), and clinical outcomes, such as angiographic evidence of graft failure, and rates of readmission and reintervention for graft occlusion, limb salvage (peripheral bypass), and mortality.

## Conclusion

The distribution of storage solutions used in Cardiothoracic and Vascular Surgery in Australia and New Zealand is not well documented in the literature. From this survey, for great saphenous veins, heparinized saline was clearly the predominant storage solution. For radial arteries, storage solutions were more diversely spread across heparinized saline, autologous blood, buffer solutions and modified buffer solutions with vasodilators. This may potentially suggest a lack of consensus with respect to radial artery storage, although responses were limited. Storage time was up to 45 min in almost half of respondents, although responses to this question were limited. Data in the literature suggests that storage with neither saline nor autologous blood is able to protect the endothelium against cold ischaemia and warm reperfusion injury. Further research is required to investigate whether ischaemic endothelial injury generates a prothrombotic state, whether different storage media can alter this state, and whether this is directly associated with clinical outcomes of interest such as early graft failure.

## Data Availability

The raw data supporting the conclusions of this article will be made available by the authors, without undue reservation.
